# Agenesis of the internal carotid artery: a rare anomaly

**DOI:** 10.1590/0100-3984.2017.0070

**Published:** 2018

**Authors:** Eduarda Lemes Dias, Luiz Gonzaga da Silveira Filho, Arthur de Freitas Ferreira

**Affiliations:** 1 Universidade Federal do Triângulo Mineiro (UFTM), Uberaba, MG, Brazil.

**Figure 1:**
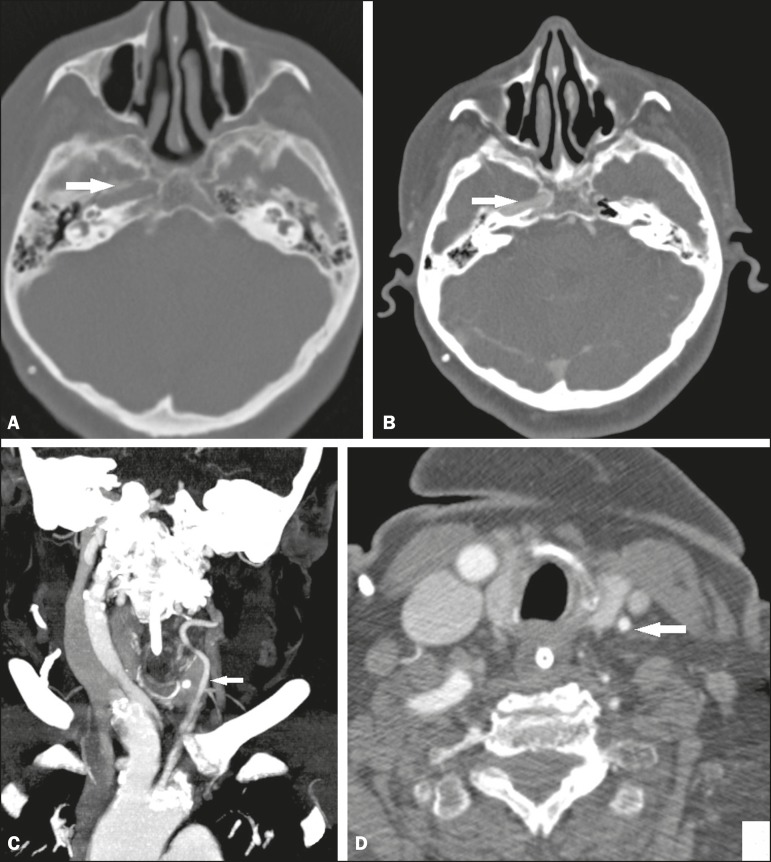
Axial CT scan of the brain (**A,B**) showing a normal right carotid canal (arrows) and absence of the left carotid canal. Coronal and axial CT angiography of the neck (**C** and **D**, respectively) showing hypoplasia of the left common carotid artery (arrows).

Dear Editor,

A 77-year-old female patient with a history of cerebral infarction episodes presented with dysphagia and dysarthria after a fall from standing height, without loss of consciousness, seven days prior. Computed tomography (CT) of the brain showed no acute ischemic infarction, although it did reveal areas of encephalomalacia, moderate microangiopathy, a reduction in brain volume, and dilation of the ventricular system. In addition, the right carotid canal was found to be absent (Figures 1A and 1B). Doppler ultrasound of the carotid arteries showed hypoplasia of the left common carotid artery and poor definition of its bifurcation. Arterial CT angiography of the head and neck showed agenesis of the left internal carotid artery (ICA), as well as ipsilateral absence of the carotid canal and hypoplasia of the common carotid artery (Figures 1C and 1D). The blood flow in the left cerebral hemisphere originated from the collateral circulation, through the anterior and left posterior communicating arteries.

ICA agenesis is a rare congenital anomaly, reported in less than 0.01% of the population. The spectrum of findings ranges from agenesis (complete absence of the ICA and carotid canal) to aplasia (absence of parts of the ICA and of the carotid canal) and hypoplasia (narrowing of the ICA and carotid canal)^(^^1^^-^^4^^)^.

Most cases of unilateral agenesis are asymptomatic, because of the collateral circulation that develops during the fetal period. Three patterns of collateral circulation have been reported. The most common type, which is similar to the one found in the present case, is the fetal variant, in which the anterior cerebral artery on the affected side is supplied by the contralateral ICA via the anterior communicating artery, and the middle cerebral artery arises from the basilar artery through the posterior communicating artery. The second pattern is the adult variant, in which the anterior and the middle cerebral arteries are supplied by the basilar artery through the posterior communicating arteries. In the third pattern, which is the rarest, transcranial anastomoses develop from the external carotid system, either from the contralateral internal carotid artery or from primitive vessels^(^^5^^,^^6^^)^.

ICA agenesis is usually an incidental finding on head and neck imaging tests, such as Doppler ultrasound, CT, or magnetic resonance imaging. However, some patients present alterations, especially when there is progression of the atherosclerotic disease. The absence of the ICA is also associated with a high incidence of cerebral aneurysms, which is approximately 25-35% in individuals without an ICA, compared with 2-4% in the general population. Less commonly, it may be associated with delayed neuropsychomotor development and agenesis of the corpus callosum, especially in patients with bilateral carotid agenesis^(^^7^^,^^8^^)^. In addition, this anomaly has major implications for the planning and execution of endarterectomy and transsphenoidal pituitary surgery. In the present case, the patient had no aneurysms or other associated malformations.

We conclude that ICA agenesis is rare and usually asymptomatic. However, careful examination of the vascular signal on magnetic resonance imaging and of carotid canals on CT, in search of stenoses (responsible for common neurological complaints), may lead to the detection of this condition, which, although asymptomatic, can be accompanied by other potentially serious diseases.
